# Epidemiological characteristics and trends of pre-hospital emergency care in Handan, China from 2011 to 2024

**DOI:** 10.3389/fpubh.2025.1651467

**Published:** 2025-09-08

**Authors:** Feng Tian, Saicong Lu, Chengcheng Bi, Xian Wang, Xu Zhang, Zhenjie Yang, Longqiang Zhang, Jie Li, Penghui Li, Haifang Zhang

**Affiliations:** ^1^School of Medicine, Hebei University of Engineering, Handan, China; ^2^Hebei Key Laboratory of Medical Data Science, Handan, China; ^3^Institute of Biomedical Information, Hebei University of Engineering, Handan, China; ^4^Public Health Research Center, Hebei University of Engineering, Handan, China; ^5^School of Information and Electrical Engineering, Hebei University of Engineering, Handan, China; ^6^Handan Emergency Rescue Command Center, Handan, China

**Keywords:** Handan, pre-hospital emergency, emergency medical service (EMS), disease spectrum, trend analysis

## Abstract

**Background:**

Pre-hospital emergency medical service (EMS) is essential in providing acute care services. This study aimed to analyze the characteristics and trends of pre-hospital EMS in Handan, China from 2011 to 2024 and provide references for the government and medical institutions to optimize EMS.

**Methods:**

Pre-hospital EMS data from 2011 to 2024 in Handan were obtained from the database of Handan Emergency Rescue Command Center. The GIS based analysis was used to reveal the spatial distribution and regional differences in pre-hospital EMS demands. The average annual growth rate (AAGR) was used to calculate the AAGR of the overall pre-hospital EMS demand and the pre-hospital diseases. Chi-square test was used to analyze gender distributions of disease spectrum regarding pre-hospital EMS. The Mann-Kendall (MK) trend test was employed to assess the trends of pre-hospital EMS demand, age distribution and disease occurrence patterns.

**Results:**

A total of 613,066 pre-hospital EMS cases from 2011 to 2024 met inclusion criteria. Over the past 14 years, Handan’s pre-hospital EMS demand increased significantly by 3.11-fold (*p* < 0.001). The percentages of EMS patients aged 61 to 70 and over 71 years old increased significantly (*p* < 0.01), in contrast, the percentage of EMS patients aged 21 to 30 and 31 to 40 years old decreased significantly (*p* < 0.01). According to ICD-10 codes, the demands per 1,000,000 people for pre-hospital care increased for 3 diseases, and injury-related diseases accounted for the largest proportion of pre-hospital emergencies. Demands because of injury-related disease, cerebrovascular diseases and pneumonia caused by unspecified organisms increased significantly (at least *p* < 0.05). As for injury-related disease, cerebrovascular diseases, heart disease and pneumonia caused by unspecified organisms, men significantly outnumbered women, although the overall demands for these diseases were high in urban areas, the demands rose quickly in peripheral counties.

**Conclusion:**

Pre-hospital EMS demand in Handan has increased substantially during the past 14 years. More medical resources should be dedicated to pre-hospital EMS due to the increased pre-hospital EMS demand. Gender, age and region distributions in diseases regarding pre-hospital EMS should also be considered.

## 1 Introduction

Pre-hospital emergency responds to the medical needs of patients with acute illnesses or injuries and has been considered as a critical component of the emergency medical service (EMS) system that ensures the safety and health of citizens (1). The world is facing two analogous megatrends that will fundamentally change the long-term emergency medical care services of its cities: urbanization and aging populations (2). Population ageing is rapidly accelerating worldwide (3). Of equal significance is the global acceleration of urbanization with more than half of the world’s population now living in cities, this is set to increase to around two-thirds by 2030 (4). The population aging and urbanization around the world presents an unprecedented set of public health challenges: chronic disease management, increased expenditure on health, long-term care, infectious disease prevention and control, pressure on medical resource allocation and so on (5). To respond to the challenges related to urban aging, China has launched the Healthy China 2030 (HC2030) plan aiming at unleashing the healthy, intellectual and vocational capacities of the older population and the whole of Chinese society (6). To do so will require filling the huge gap in characteristics and trends of the long-term emergency medical care services, which involved a high prevalence of comorbidity and multimorbidity, with consistent health disparities among age groups, between men and women, and between rural and urban areas (7). Following the standard of the “Notice of the State Council on Adjusting the Standards for Urban Scale” (8), among the 105 large cities, there are 7 megacities, 14 super large cities, 14 Type-I large cities, and 70 Type-II large cities, according to China’s 7th National Population Census (9). Although Type-II large cities account for the largest proportion at 67%, the existing studies were mainly characterized the trends of pre-hospital EMS in megacities, super large cities or Type-I large cities (8) of China, such as Beijing (1, 10), Hangzhou (11), Chengdu (12). A study conducted in Berlin, Germany revealed that younger age was the strongest predictor for low-acuity calls, which were not the primary driver of increased EMS utilisation in Berlin (13). According to studies conducted in Vienna, Austria, incidence and case load of severe penetrating chest trauma increased, and potentially life-saving invasive procedures were only reluctantly applied (14), the older adult people ≥65 years in Vienna shows higher EMS response rates than younger adults, which should trigger further research and the development of solutions, such as specific response units dedicated to older adult people (15). In recent discourse on pre-hospital EMS, megacities, super large cities or Type-I large cities have frequently obtained a disproportionate amount of attention over other sizes of cities worldwide. Here, it was argued that a focus on Type-II large cities, medium-sized cities or small cities was crucial to respecting the life, making full use of medical resources and honoring social equality principle, not only because they were home to at least half of the world’s population but because they also offer great potential for sustainable improvement of pre-hospital EMS. To date, few studies focused on the comprehensive, long-term analysis of pre-hospital EMS demand in Type-II large cities, medium-sized cities or small cities, particularly with an integrated view of age distribution, disease spectrum, and demographic shifts. This situation would compromise the efficiency and quality of the prehospital emergency care continuum.

Using Handan, a representative Type-II large city (8) with a relative large economic aggregate in North China, as a case study, this study sought to investigate long-term characteristics and trends of pre-hospital emergency care in the context of urban aging. The urban aging-related problems, such as chronic health care, public financial pressure, and labor shortage, as well as public health emergencies and road congestion, have brought severe shocks to pre-hospital emergency services and grave challenges to medical deployment capabilities in Handan. To date, little research has been designed to investigate Handan’s pre-hospital EMS, which is warranted for integrating regional medical resources, as well as for accelerating the establishment of an early warning linkage system between prehospital and in-hospital emergency services and improving multidisciplinary cooperation for urgent treatment. The main objectives of this study were to describe real-world characteristics of EMS demand in Handan for 14 years, to reflect population aging trends through detailed age group analysis, to explore how the disease spectrum of EMS has evolved over time, and to analyze how gender, age, and regional disparities are associated with changes in EMS relevant diseases. The conclusions drawn from this study can offer a theoretical basis for the government and medical institutions to improve the quality of emergency care in Handan, which we believed, were likely applicable to other Type-II large cities, due to the patterns observed in this study are relevant to other Type-II large cities that underwent similar socioeconomic changes in the world.

## 2 Materials and methods

### 2.1 Study design

This study was a retrospective observational study that analyzed the characteristics and trends of pre-hospital EMS in Handan, China from 2011 to 2024. Specifically, data for this study were sourced from the database of Handan Emergency Rescue Command Center, Hebei province, China. For the purpose of spatial analysis, we divided the study area into two categories: Urban Areas and County Towns, based on the historical administrative division of Handan. Urban Areas refer to the three traditional central districts of Handan—Hanshan, Congtai, and Fuxing, which historically represent the city’s main urban areas. County Towns include all other administrative regions under the jurisdiction of Handan, such as counties and county level districts beyond the three central districts. The study received ethics approval from the Ethics Committee of Hebei University of Engineering (BER-YXY-2024027), in accordance with the Declaration of Helsinki. The Ethics Committee of Hebei University of Engineering waived the requirement for informed consent due to the retrospective nature of the study. All methods were performed in accordance with the relevant guidelines and regulations. The data used in this study were anonymous.

### 2.2 Study setting

Handan is a representative Type-II large city with a large economic aggregate located in the south of Hebei province at the junction of Hebei, Henan, Shanxi and Shandong provinces with an area of 12,066 km^2^. By the end of 2024, Handan’s GDP had reached 470.43 billion Chinese Yuan, reflecting 14-year increases of 68.77% (16, 17). Cities with a permanent population of over 10 million in urban areas are considered as megacities; Cities with a permanent population of 5 million to 10 million in urban areas are classified as super large cities; Cities with a permanent population of 1 million to 5 million in urban areas are considered as large cities, among which cities with a population of 3 million to 5 million are classified as Type I large cities and cities with a population of 1 million to 3 million are Type II large cities; Cities with a permanent population of 500,000 to 1 million in urban areas are classified as medium-sized cities; Cities with a permanent population of less than 500,000 in urban areas are considered as small cities, among which cities with a population of 200,000 to 500,000 are classified as Type I small cities, and cities with a population of less than 200,000 are classified as Type II small cities (8). According to China’s 7th National Population Census (9), Handan was classified as Type II large city, with a permanent population of 1.94 million in urban areas. The total population of Handan had reached 9.18 million as of 2024, and the growth rate of older adult population aged 60 and above in Handan experienced an increase for 14 years, increasing from 11.37 to 19.56%, which means Handan has already entered the aging society (17, 18). The urbanization rate of permanent population in Handan has seen significant growth over the years. In 2024, the urbanization rate has reached 62.10%, with 5.70 million people living in urban areas. This marks a substantial increase from 46.67% by the end of 2011, reflecting Handan’s rapid urbanization process (16, 17). Since the establishment of Handan Emergency Rescue Command Center (emergency telephone number: 120) (19) in 2007, it has adopted a similar “command mode,” as has Guangzhou Medical Emergency Command Center (20), and has been responsible for commanding and dispatching pre-hospital emergency vehicles and personnel in Handan.

### 2.3 Data collection

**Figure 1:**
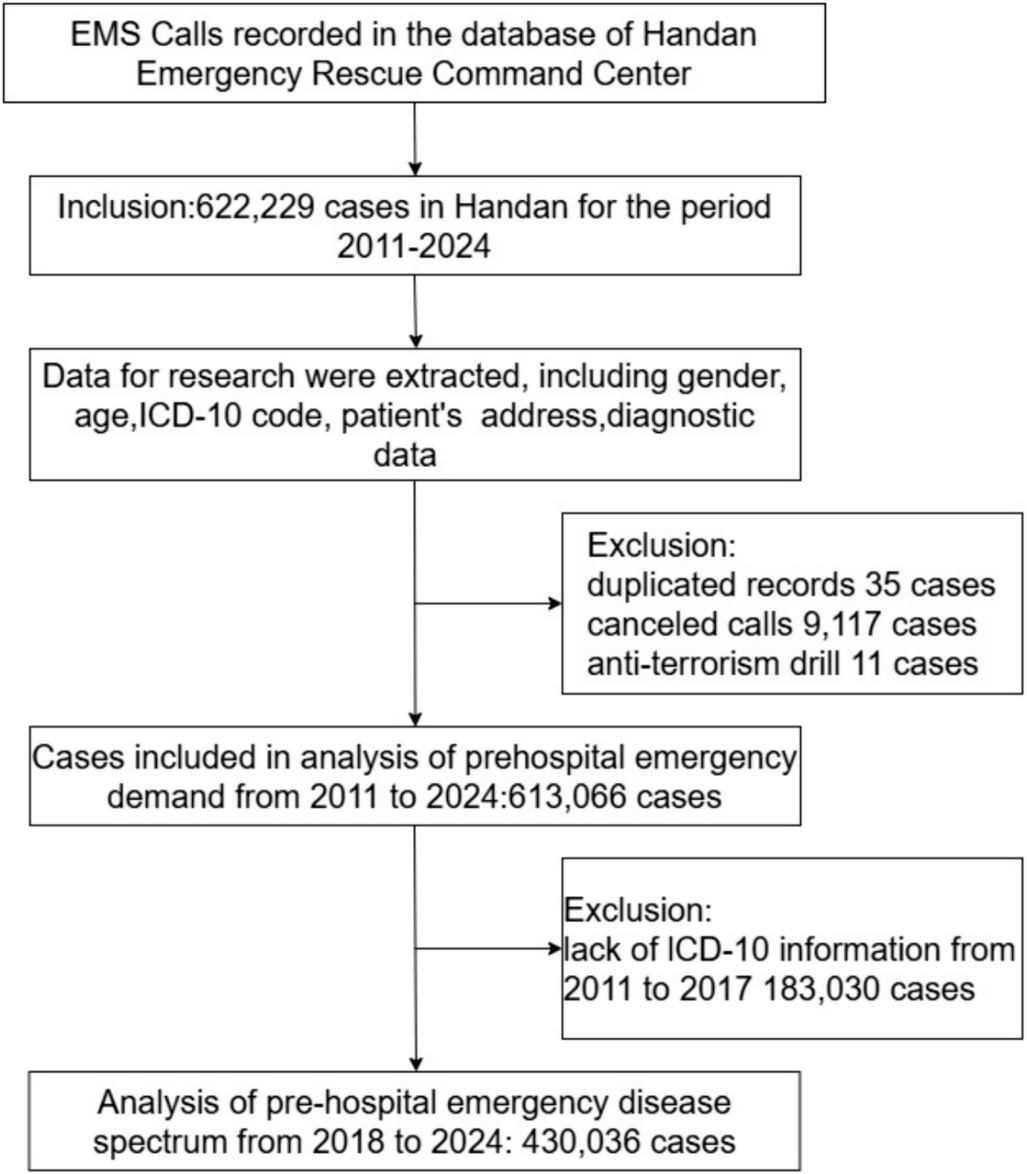
Flowchart of case inclusion and exclusion.

The pre-hospital data recorded by emergency medical dispatchers was collected from the database of Handan Emergency Rescue Command Center, Hebei province, China. The population numbers we used were obtained from the Handan Municipal Bureau of Statistics. Information regarding these cases included gender, age, diagnosis data, International Classification of Diseases, 10th edition (ICD-10) code, patients’ address. Figure 1 summarizes the data sources and the inclusion/exclusion criteria of the pre-hospital emergency cases. The pre-hospital emergency cases in Handan for the period 2011–2024 were included. Duplicate records were identified and removed to ensure data reliability. Canceled emergency calls were excluded, as they did not contribute to the assessment of pre-hospital emergency medical service utilization. Anti-terrorism drill cases were also omitted due to the fact that they were not emergency rescue missions. When calculating pre-hospital EMS demand rates (per 1,000,000 people) and gender-standardized incidence rates per 1,000,000 people, we used population numbers obtained from the Handan Bureau of Statistics (17).

### 2.4 Geographic information system (GIS) analysis

The GIS based analysis was carried out to reveal the spatial distribution and regional differences in pre-hospital EMS demands in Handan. The address of each record was classified by county-level division based on Gaode HTTP API. Statistics and visualization were implemented using the Python package Pyecharts (Version 2.0.1).

### 2.5 ICD-10 classification

The pre-hospital emergency diseases from 2018 to 2024 were classified by clinicians according to the ICD-10 codes. To analyze the disease spectrum regarding pre-hospital EMS, the frequency of the first- and second categories of ICD-10 were calculated. Then, the annual and gender variation of disease spectrum in Handan were evaluated by using the Python packages of import pandas as pd. (Version 1.5.1), import matplotlib.pyplot as plt (Version 3.6.2), and import numpy as np (Version 1.26.4).

### 2.6 Statistical analyses

The enumeration data in the study were expressed as frequency and percentage, and were analyzed by chi-square test. The average annual growth rate (AAGR) expressed in terms of a geometric growth expressed as (X_n_/X_0_)(1/n)-1 (where X_0_ is the value of the baseline and X_n_ is the value of the nth year) (21), was used to calculate the AAGR of the overall pre-hospital EMS demand and the pre-hospital EMS diseases. The two-sided Mann-Kendall (MK) trend test was employed to assess the trends of pre-hospital EMS demand, age distribution and disease occurrence patterns. Python (Version 3.10.7) and IBM SPSS Statistics software version 25 were used for data analysis, and *p* < 0.05 was considered statistically significant.

## 3 Results

### 3.1 Growth of pre-hospital EMS demand from 2011 to 2024

From 2011 to 2024, 622,229 records were contained in the database of Handan Emergency Rescue Command Center we used in this study. We excluded duplicate records (*n* = 35), cases in which patients canceled emergency requests (*n* = 9,117), and anti-terrorism drill cases that were not emergency rescue missions (*n* = 11). A final total of 613,066 cases were used for analyzed for the analysis of pre-hospital EMS demand, among which, 183,030 cases were collected without ICD-10 code from 2011 to 2017 (Figure 1). Since 2018, with the information construction of pre-hospital emergency in Handan, a total of 430,036 cases with diagnostic information were collected from 2018 to 2024, labelled by clinicians according to ICD-10 and analyzed for disease spectrum and disease occurrence patterns (Figure 1).

**Figure 2:**
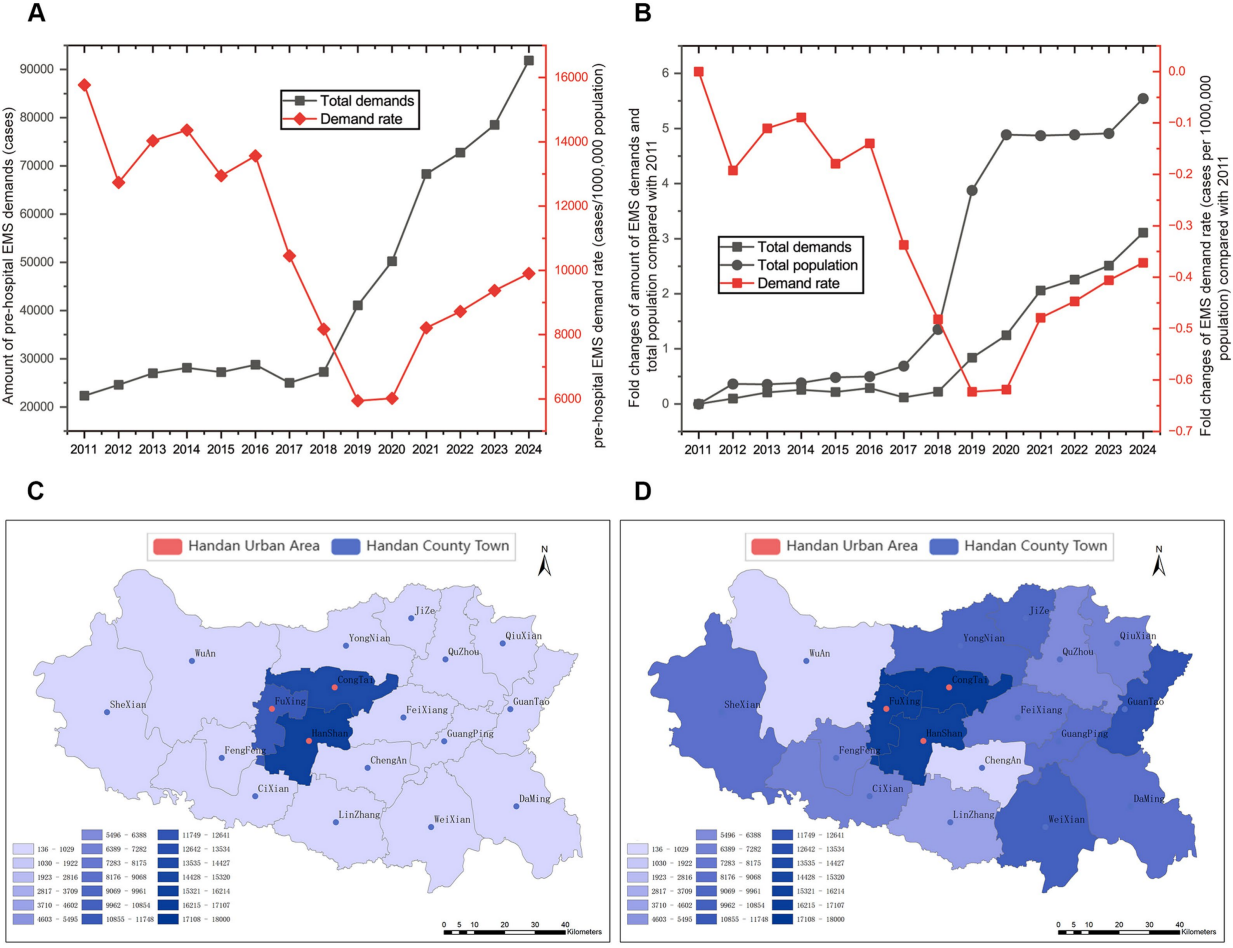
Changes and distribution of pre-hospital emergency medical service (EMS) demand. Changes of pre-hospital EMS demands and pre-hospital EMS demands per 1,000,000 people **(A)** and the fold change for pre-hospital EMS demands, total population and pre-hospital EMS demands per 1,000,000 people compared with 2011 **(B)** during 2011 to 2024. The distribution of pre-hospital EMS demands per 1,000,000 people in 2011 **(C)** and 2024 **(D)** based on GIS. Urban areas and county districts are marked as red and blue, respectively.

As a whole, Handan’s pre-hospital EMS demand increased significantly by 3.11-fold (*p* < 0.001), from 22,353 cases in 2011 to 91,862 cases in 2024 with an AAGR of 11.48% (Figure 2A). After 6 years’ fluctuation, the total pre-hospital EMS demand increased by 2.67-fold from 25,012 cases in 2017 to 91,862 cases in 2024 with an AAGR of 20.42% (*p* < 0.01, Figure 2B). As for the pre-hospital EMS demand per 1,000,000 people in Handan, it first fluctuated, then displayed a decrease marked decline from 2016 to 2019, and finally increased by 66.56% from 5,946.62 per 1,000,000 people in 2019 to 9,904.86 per 1,000,000 people in 2024 (*p* < 0.01, Figures 2A,B). According the GIS analysis, the pre-hospital EMS demand of each county increased rapidly, and with the spread of pre-hospital EMS network, the farther counties demand number increased faster (Figures 2C,D).

### 3.2 The age distribution of pre-hospital EMS patients

To determine the age distribution of pre-hospital EMS patients, the percentages of EMS patients by age group (10-year intervals) were calculated. From 2018 to 2024, the percentages of EMS patients aged 61 to 70 and over 71 years old increased significantly (*p* < 0.01, *p* < 0.01, respectively), in contrast, the percentage of EMS patients aged 21 to 30 and 31 to 40 years old decreased significantly (*p* < 0.01, *p* < 0.01, respectively, Table 1). There was no significant increase (*p* > 0.05) in the percentages of EMS patients aged 0 to 10, 11 to 20, 41 to 50, and 51 to 60 years old, and the age distribution of these patients fluctuated wildly (Table 1).

**Table 1:** The age percentages trends of the pre-hospital emergency medical service patients from 2018 to 2024.

Age groups	2018 N (%)	2019 N (%)	2020 N (%)	2021 N (%)	2022 N (%)	2023 N (%)	2024 N (%)	*p-*value	Trend
0 ~ 10	176.05 (2.15%)	154.11 (2.59%)	142.33 (2.37%)	275.88 (3.36%)	265.14 (3.04%)	238.42 (2.54%)	243.03 (2.45%)	0.548 (0.764)	- (−)
11 ~ 20	704.22 (8.62%)	481.00 (8.09%)	459.21 (7.63%)	649.96 (7.91%)	614.15 (7.04%)	719.31 (7.67%)	830.13 (8.38%)	0.230 (0.548)	- (−)
21 ~ 30	745.31 (9.12%)	502.27 (8.45%)	474.19 (7.88%)	614.98 (7.49%)	586.47 (6.73%)	621.06 (6.63%)	661.93 (6.68%)	0.548 (0.007)	- (↓)
31 ~ 40	1484.32 (18.16%)	1051.85 (17.69%)	1052.01 (17.49%)	1314.11 (16.00%)	1261.60 (14.47%)	1336.67 (14.26%)	1299.70 (13.12%)	0.764 (0.003)	- (↓)
41 ~ 50	1013.74 (12.40%)	693.86 (11.67%)	690.80 (11.48%)	942.19 (11.47%)	896.54 (10.28%)	1054.43 (11.25%)	1148.53 (11.60%)	0.230 (0.133)	- (−)
51 ~ 60	1642.38 (20.09%)	1193.23 (20.07%)	1242.02 (20.65%)	1658.39 (20.19%)	1699.98 (19.50%)	1691.73 (18.05%)	1662.96 (16.79%)	0.133 (0.072)	- (−)
61 ~ 70	948.36 (11.60%)	791.97 (13.32%)	833.00 (13.85%)	1152.67 (14.03%)	1291.07 (14.81%)	1458.08 (15.56%)	1615.41 (16.31%)	0.016 (0.003)	↑ (↑)
>70	1459.72 (17.86%)	1078.34 (18.13%)	1122.33 (18.66%)	1606.82 (19.56%)	2103.50 (24.13%)	2252.49 (24.03%)	2443.17 (24.67%)	0.016 (0.007)	↑ (↑)

N, number of pre-hospital emergency medical service cases per 1,000,000 people.

### 3.3 Disease spectrum regarding pre-hospital EMS

**Figure 3:**
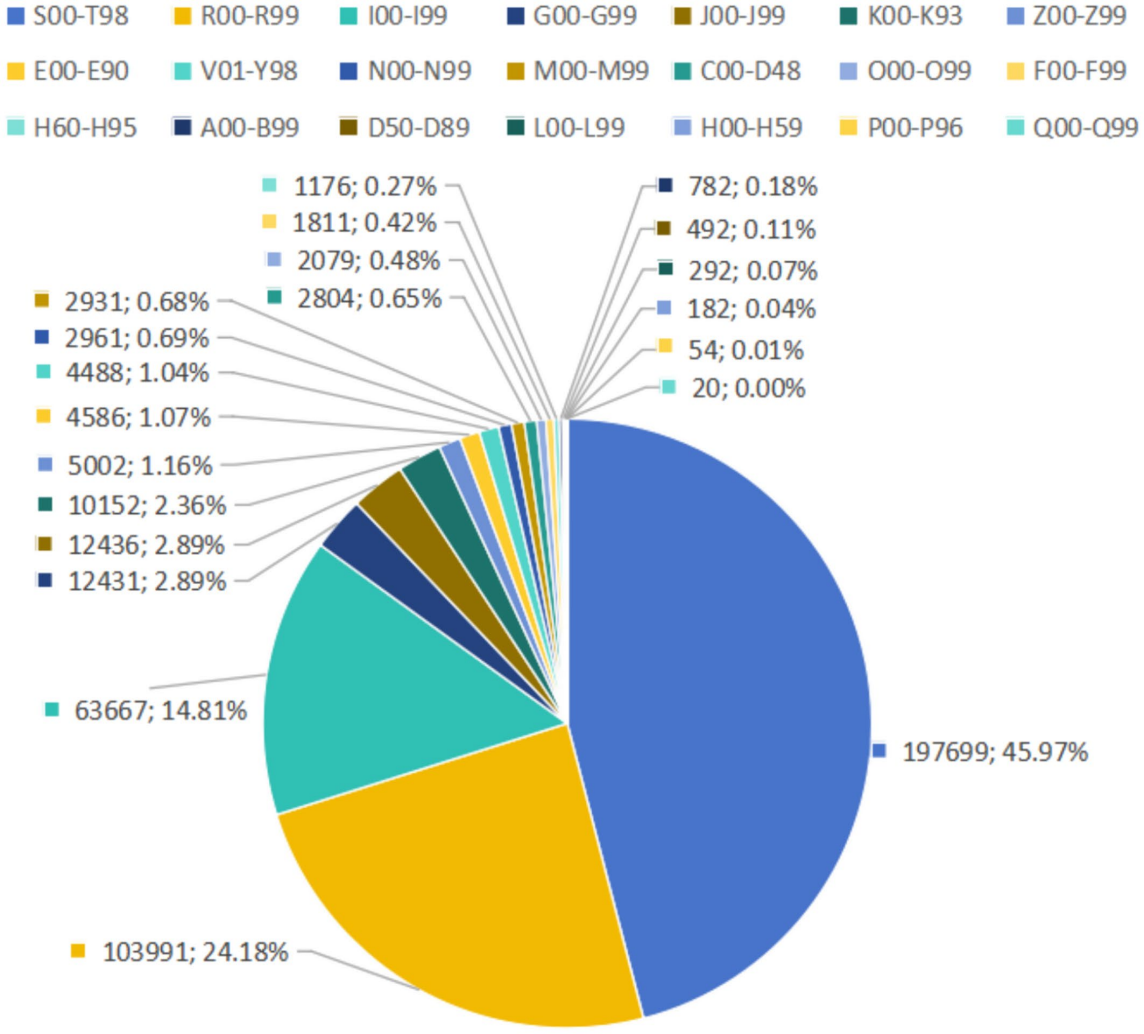
The composition ratio of different pre-hospital emergency medical service diseases from 2018 to 2024.

**Figure 4:**
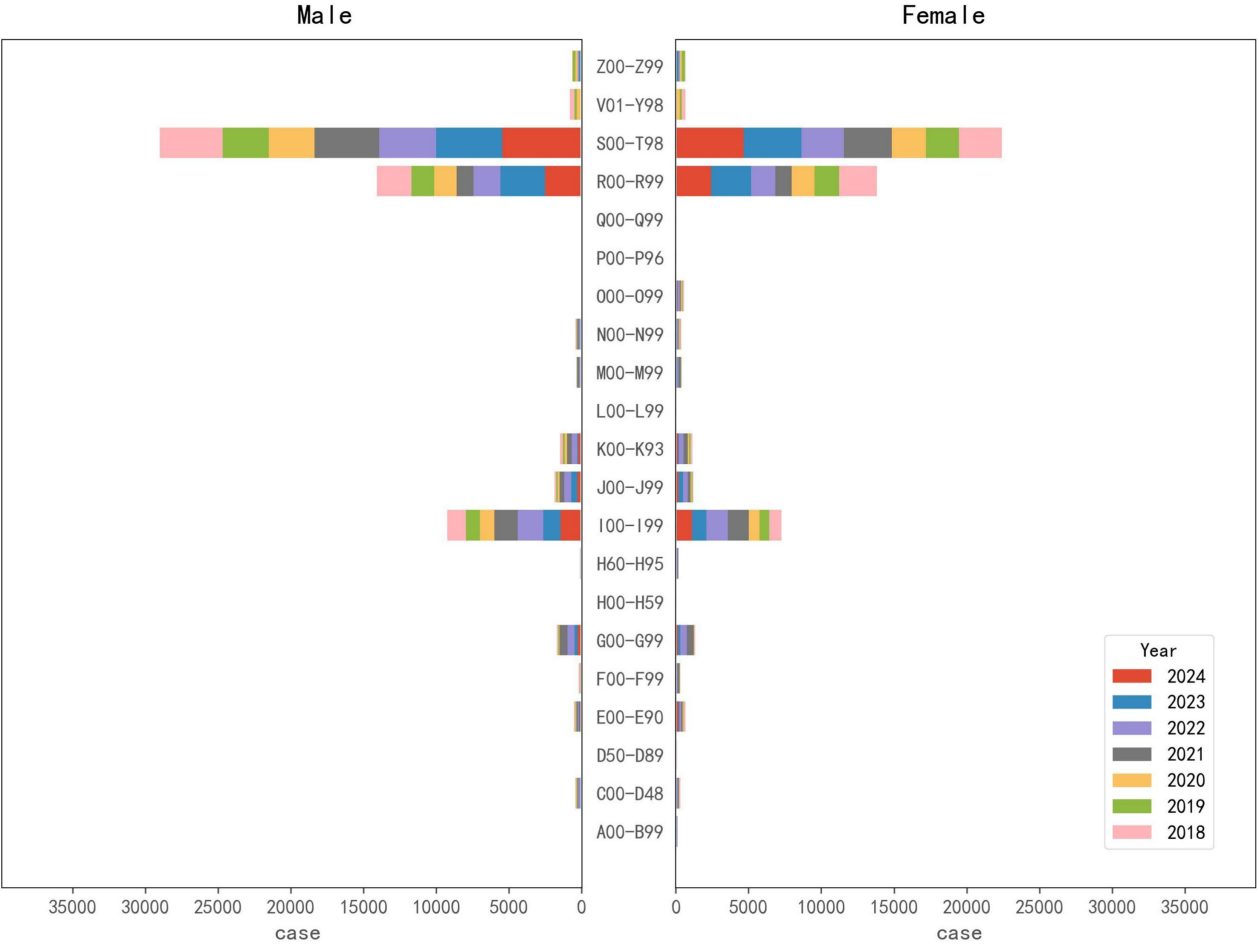
The gender distribution of different pre-hospital emergency medical service diseases from 2018 to 2024.

From 2018 to 2024, the top five pre-hospital EMS diseases were injury, poisoning and certain other consequences of external causes (197,699 cases, 45.97%), symptoms, signs and abnormal clinical and laboratory findings, not elsewhere classified (103,991 cases, 24.18%), diseases of the circulatory system (63,667 cases, 14.81%), diseases of the respiratory system (12,436, 2.89%), and diseases of the nervous system (12,431 cases, 2.89%) (Figure 3 and Table 2). There were significant increases in 6 ICD-10 categories, among which, injury, poisoning and certain other consequences of external causes increased significantly over time with an AAGR of 25.29%, and the pre-hospital EMS demand in 15 categories remained fluctuated (Table 3). As for the percentage change trend of diseases, there were only 3 ICD-10 categories that exhibited significant increases, and the other categories remained fluctuated (Table 3). Chi-square test was used to analyze gender distributions of disease spectrum regarding pre-hospital EMS. Among the pre-hospital EMS patients, 55.15% were males, 44.85% were females (Figure 4 and Table 2). The disease distributions showed statistically significant difference between male and female (X^2^ = 4339.21, *p* < 0.001).

**Table 2:** Disease spectrum regarding pre-hospital emergency medical service by gender in Handan.

Coding for diseases	Types of diseases	Male *N* (%)	Female *N* (%)	Total *N* (%)
A00-B99	Certain infectious and parasitic diseases	335 (42.84%)	447 (57.16%)	782 (100.00%)
C00-D48	Neoplasms	1,698 (60.56%)	1,106 (39.44%)	2,804 (100.00%)
D50-D89	Diseases of the blood and blood-forming organs and certain disorders involving the immune mechanism	222 (45.12%)	270 (54.88%)	492 (100.00%)
E00-E90	Endocrine, nutritional and metabolic diseases	2067 (45.07%)	2,519 (54.93%)	4,586 (100.00%)
F00-F99	Mental and behavioral disorders	690 (38.10%)	1,121 (61.90%)	1811 (100.00%)
G00-G99	Diseases of the nervous system	7,061 (56.80%)	5,370 (43.20%)	12,431 (100.00%)
H00-H59	Diseases of the eye and adnexa	94 (51.65%)	88 (48.35%)	182 (100.00%)
H60-H95	Diseases of the ear and mastoid process	404 (34.35%)	772 (65.65%)	1,176 (100.00%)
I00-I99	Diseases of the circulatory system	36,068 (56.65%)	27,599 (43.35%)	63,667 (100.00%)
J00-J99	Diseases of the respiratory system	7,642 (61.45%)	4,794 (38.55%)	12,436 (100.00%)
K00-K93	Diseases of the digestive system	5,779 (56.92%)	4,373 (43.08%)	10,152 (100.00%)
L00-L99	Diseases of the skin and subcutaneous tissue	154 (52.74%)	138 (47.26%)	292 (100.00%)
M00-M99	Diseases of the musculoskeletal system and connective tissue	1,396 (47.63%)	1,535 (52.37%)	2,931 (100.00%)
N00-N99	Diseases of the genitourinary system	1,652 (55.79%)	1,309 (44.21%)	2,961 (100.00%)
O00-O99	Pregnancy, childbirth and the puerperium	14^*^ (0.67%)	2065 (99.33%)	2079 (100.00%)
P00-P96	Certain conditions originating in the perinatal period	26 (48.15%)	28 (51.85%)	54 (100.00%)
Q00-Q99	Congenital malformations, deformations and chromosomal abnormalities	6 (30.00%)	14 (70.00%)	20 (100.00%)
R00-R99	Symptoms, signs and abnormal clinical and laboratory findings, not elsewhere classified	53,957 (51.89%)	50,034 (48.11%)	103,991 (100.00%)
S00-T98	Injury, poisoning and certain other consequences of external causes	112,822 (57.07%)	84,877 (42.93%)	197,699 (100.00%)
V01-Y98	External causes of morbidity and mortality	2,508 (55.88%)	1980 (44.12%)	4,488 (100.00%)
Z00-Z99	Factors influencing health status and contact with health services	2,551 (51.00%)	2,451 (49.00%)	5,002(100.00%)
Total	-	237,146 (55.15%)	192,890 (44.85%)	430,036 (100.00%)

N, number of pre-hospital emergency medical service cases. *In 14 rare cases, emergency telephone operators used the male newborns to describe the cases of delivery in the category of O00-O99.

**Table 3:** Trends of diseases in prehospital emergency in Handan.

Coding for diseases	Types of diseases	2018 N (%)	2019 N (%)	2020 N (%)	2021 N (%)	2022 N (%)	2023 N (%)	2024 N (%)	*p-*value	Trend
A00-B99	Certain infectious and parasitic diseases	1.20 (0.01%)	0.14 (0.00%)	0.60 (0.01%)	3.49 (0.04%)	5.27 (0.06%)	41.31 (0.44%)	38.06 (0.38%)	0.036 (0.036)	↑ (↑)
C00-D48	Neoplasms	52.49 (0.64%)	26.05 (0.44%)	31.39 (0.52%)	75.61 (0.92%)	85.66 (0.98%)	49.67 (0.53%)	46.04 (0.46%)	0.764 (1.000)	- (−)
D50-D89	Diseases of the blood and blood-forming organs and certain disorders involving the immune mechanism	6.00 (0.07%)	3.91 (0.07%)	3.47 (0.06%)	11.66 (0.14%)	14.26 (0.16%)	13.01 (0.14%)	9.81 (0.10%)	0.368 (0.548)	- (−)
E00-E90	Endocrine, nutritional and metabolic diseases	81.58 (1.00%)	43.12 (0.73%)	54.15 (0.90%)	91.60 (1.12%)	97.05 (1.11%)	70.08 (0.75%)	151.49 (1.53%)	0.133 (0.368)	- (−)
F00-F99	Mental and behavioral disorders	55.49 (0.68%)	7.09 (0.12%)	23.36 (0.39%)	72.73 (0.89%)	54.27 (0.62%)	0.00 (0.00%)	34.93 (0.35%)	0.764 (0.548)	- (−)
G00-G99	Diseases of the nervous system	68.98 (0.84%)	34.15 (0.57%)	42.77 (0.71%)	504.63 (6.41%)	452.28 (5.19%)	207.97 (2.22%)	204.11 (2.06%)	0.548 (0.548)	- (−)
H00-H59	Diseases of the eye and adnexa	0.90 (0.01%)	0.87 (0.01%)	0.12 (0.00%)	0.12 (0.00%)	1.80 (0.02%)	10.39 (0.11%)	7.44 (0.08%)	0.230 (0.230)	- (−)
H60-H95	Diseases of the ear and mastoid process	0.30 (0.00%)	0.29 (0.00%)	0.36 (0.01%)	53.61 (0.65%)	55.59 (0.64%)	0.00 (0.00%)	28.03 (0.28%)	0.548 (0.548)	- (−)
I00-I99	Diseases of the circulatory system	1056.03 (12.92%)	824.81 (13.87%)	861.40 (14.32%)	1526.76 (18.59%)	1633.61 (18.74%)	1089.53 (11.63%)	1271.67 (12.84%)	0.230 (1.000)	- (−)
J00-J99	Diseases of the respiratory system	110.07 (1.35%)	75.25 (1.27%)	85.78 (1.43%)	263.74 (3.21%)	410.35 (4.71%)	363.42 (3.88%)	233.98 (2.36%)	0.230 (0.133)	- (−)
K00-K93	Diseases of the digestive system	155.36 (1.90%)	112.87 (1.90%)	135.98 (2.26%)	302.80 (3.69%)	342.90 (3.93%)	70.20 (0.75%)	188.69 (1.91%)	0.764 (0.764)	- (−)
L00-L99	Diseases of the skin and subcutaneous tissue	1.50 (0.02%)	2.17 (0.04%)	1.44 (0.02%)	4.81 (0.06%)	5.27 (0.06%)	7.40 (0.08%)	12.29 (0.12%)	0.016 (0.007)	↑ (↑)
M00-M99	Diseases of the musculoskeletal system and connective tissue	36.29 (0.44%)	13.31 (0.22%)	8.27 (0.14%)	138.48 (1.69%)	95.49 (1.10%)	20.42 (0.22%)	57.04 (0.58%)	0.764 (1.000)	- (−)
N00-N99	Diseases of the genitourinary system	57.89 (0.71%)	38.93 (0.65%)	46.84 (0.78%)	62.51 (0.76%)	92.49 (1.06%)	49.78 (0.53%)	43.02 (0.43%)	1.000 (0.548)	- (−)
O00-O99	Pregnancy, childbirth and the puerperium	41.99 (0.51%)	30.82 (0.52%)	28.03 (0.47%)	44.24 (0.54%)	66.85 (0.77%)	17.55 (0.19%)	45.18 (0.46%)	0.764 (0.764)	- (−)
P00-P96	Certain conditions originating in the perinatal period	1.80 (0.02%)	0.58 (0.01%)	0.84 (0.01%)	0.12 (0.00%)	0.36 (0.00%)	0.84 (0.01%)	2.80 (0.03%)	1.000 (1.000)	- (−)
Q00-Q99	Congenital malformations, deformations and chromosomal abnormalities	0.00 (0.00%)	0.00 (0.00%)	0.12 (0.00%)	0.00 (0.00%)	0.36 (0.00%)	1.19 (0.01%)	0.65 (0.01%)	0.042 (0.042)	↑ (↑)
R00-R99	Symptoms, signs and abnormal clinical and laboratory findings, not elsewhere classified	2482.46 (30.37%)	1625.75 (27.34%)	1545.25 (25.69%)	1139.93 (13.88%)	1775.82 (20.37%)	2926.44 (31.22%)	2454.49 (24.78%)	0.764 (0.548)	- (−)
S00-T98	Injury, poisoning and certain other consequences of external causes	3645.26 (44.60%)	2750.68 (46.26%)	2742.70 (45.59%)	3887.64 (47.32%)	3429.08 (39.33%)	4259.28 (45.45%)	5068.23 (51.17%)	0.133 (0.548)	- (−)
V01-Y98	External causes of morbidity and mortality	279.53 (3.42%)	158.31 (2.66%)	250.63 (4.17%)	29.81 (0.36%)	0.48 (0.01%)	6.45 (0.07%)	6.90 (0.07%)	0.072 (0.133)	- (−)
Z00-Z99	Factors influencing health status and contact with health services	38.99 (0.48%)	197.52 (3.32%)	152.39 (2.53%)	0.72 (0.01%)	99.20 (1.14%)	167.26 (1.78%)	0.00 (0.00%)	0.548 (0.368)	- (−)

N number of pre-hospital emergency medical service cases per 1,000,000 people.

### 3.4 Gender, age and region changes in diseases regarding pre-hospital EMS

**Figure 5:**
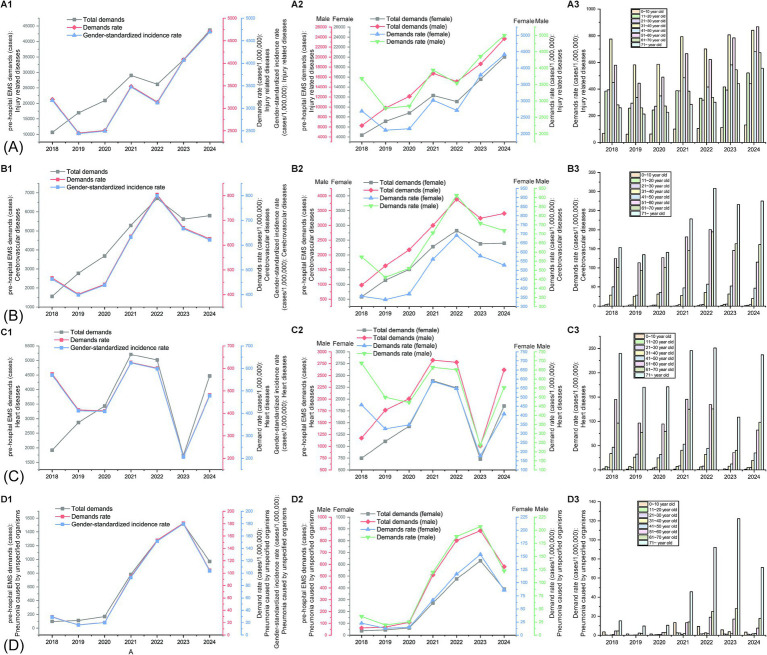
Gender and age changes in diseases regarding pre-hospital emergency medical service from 2018 to 2024. **(A)** Changes in the pre-hospital demand related to injury-related diseases: (A1), amount of pre-hospital demands, pre-hospital demands per 1,000,000 people, and gender-standardized incidence rates of injury-related diseases per 1,000,000 people; (A2) gender changes in amount of pre-hospital demands and pre-hospital demands per 1,000,000 people; (A3) age changes in pre-hospital demands per 1,000,000 people for injury-related diseases. **(B)** Changes in the pre-hospital demand related to cerebrovascular diseases: (B1), number of pre-hospital cases, pre-hospital cases per 1,000,000 people, and gender-standardized incidence rates of cerebrovascular diseases per 1,000,000 people; (B2) gender changes in amount of pre-hospital demands and pre-hospital demands per 1,000,000 people; (B3) age changes in pre-hospital demands per 1,000,000 people for cerebrovascular diseases. **(C)** Changes in the pre-hospital demand related to heart diseases: (C1), amount of pre-hospital demands, pre-hospital demands per 1,000,000 people, and gender-standardized incidence rates of heart diseases per 1,000,000 people; (C2) gender changes in amount of pre-hospital demands and pre-hospital demands per 1,000,000 people; (C3) age changes in pre-hospital demands per 1,000,000 people for heart diseases. **(D)** Changes in the pre-hospital demand related to pneumonia caused by unspecified organisms: (D1), amount of pre-hospital demands, pre-hospital demands per 1,000,000 people, and gender-standardized incidence rates of pneumonia caused by unspecified organisms per 1,000,000 people; (D2) gender changes in amount of pre-hospital demands and pre-hospital demands per 1,000,000 people; (D3) age changes in pre-hospital demands per 1,000,000 people for pneumonia caused by unspecified organisms.

**Figure 6:**
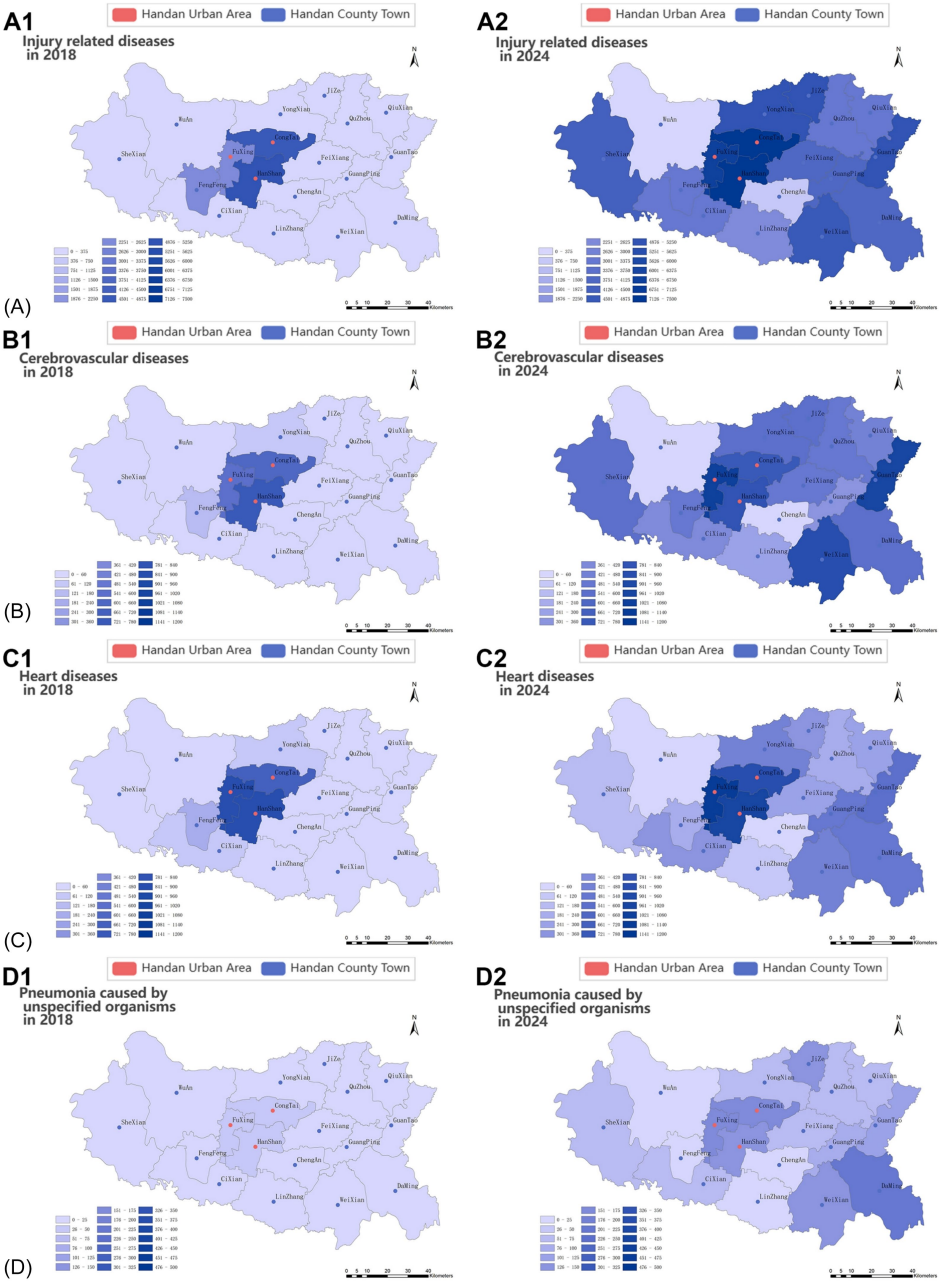
Region changes in diseases regarding pre-hospital emergency medical service. **(A)** Heat map of the pre-hospital demands per 1,000,000 people related to injury-related disease in 2018 (A1) and 2024 (A2); **(B)** heat map of the pre-hospital demands per 1,000,000 people related to cerebrovascular diseases in 2018 (B1) and 2024 (B2); **(C)** heat map of the pre-hospital demands per 1,000,000 people related to heart disease in 2018 (C1) and 2024 (C2); **(D)** heat map of the pre-hospital demands per 1,000,000 people related to pneumonia caused by unspecified organisms in 2018 (D1) and 2024 (D2).

In 2018, there were 10,660 injury-related disease cases regarding pre-hospital demand, reaching 43,694 (accounting for 47.56% of pre-hospital care) in 2024, which showed a significant growth (*p* < 0.01, Figure 5A1). Likewise, the demands per 1,000,000 people and gender-standardized incidence rates per 1,000,000 people for injury-related diseases exhibited a significant growth trend from 2019 to 2024 (*p* < 0.05, Figure 5A1). The demands per 1,000,000 people for injury-related diseases in peripheral counties (such as Yongnian, Guantao, and Weixian) were lower than that in the urban areas of Handan, but it increased rapidly (Figures 6A1,A2). Demands and demands per 1,000,000 people for injury-related diseases in males were significantly higher than that of females (X^2^ = 236.20 and 62.77, *p* < 0.001 and 0.001, respectively), (Figure 5A2). The demand rate of patients aged 31 to 40, 51 to 60, and 41 to 50 years old took the top three places in terms of injury-related diseases regarding pre-hospital demand per 1,000,000 people, followed by patients aged 61 to 70 and 11 to 20 years old (Figure 5A3). Patients aged 21 to 30 and 31 to 40 years old displayed significant decreasing percentage trends (*p* < 0.01 and *p* < 0.01, respectively), whereas patients aged 61 to 70 and over 71 years old displayed significant increasing percentage trends (*p* < 0.05 and *p* < 0.05, respectively, Supplementary Table 1).

Demands because of cerebrovascular diseases increased significantly (*p* < 0.05), from 1,560 cases in 2018 to 5,798 cases in 2024 (Figure 5B1), accounting for 6.31% of pre-hospital care in 2024. Likewise, the demands per 1,000,000 people and gender-standardized incidence rates per 1,000,000 people for cerebrovascular diseases showed a growth trend, but no significant trend was detected (*p* > 0.05, Figure 5B1). In contrast, demands, demands per 1,000,000 people, and gender-standardized incidence rates per 1,000,000 people because of heart diseases fluctuated wildly from 2018 to 2024 (*p* > 0.05) (Figure 5C1). Regional variation of the demands per 1,000,000 people for cerebrovascular diseases and heart disease was the same as injury-related diseases in Handan, that is, the overall demands per 1,000,000 people were high in the urban areas, but it rose at a quicker pace in peripheral counties (Figures 6B1,B2,C1,C2). Demands for cerebrovascular diseases and heart diseases in males were significantly higher than that of females (X^2^ = 19.90 and 65.24, *p* < 0.01 and 0.001, respectively, Figures 5B2,C2). Moreover, demands per 1,000,000 people for heart diseases in males were significantly higher than that of females (X^2^ = 19.67, *p* < 0.01, Figure 5C2). Patients aged 61 to 70 and over 71 years old displayed significant increasing percentage trends in cerebrovascular diseases (*p* < 0.05 and *p* < 0.05, respectively, Figure 5B3 and Supplementary Table 2). Although patients aged 61 to 70 displayed significant increasing percentage trends in heart diseases (*p* < 0.05), patients aged 51 to 60 displayed significant decreasing percentage trends in heart diseases (*p* < 0.01, Figure 5C3 and Supplementary Table 3).

Demands for pneumonia caused by unspecified organisms increased significantly (*p* < 0.05), was 98 in 2018 and 970 in 2024 (accounting for 1.06% of pre-hospital care), but remained at relatively low levels (Figure 5D1). Likewise, the demands per 1,000,000 people and gender-standardized incidence rates per 1,000,000 people for pneumonia caused by unspecified organisms exhibited a growth trend, but remained at relatively low levels and no significant trend was detected (*p* > 0.05, Figure 5D1). Although the demands per 1,000,000 people for pneumonia caused by unspecified organisms were rapidly growing across Handan, the overall demands per 1,000,000 people for pre-hospital EMS was low in both the urban areas and peripheral counties, because of pneumonia caused by unspecified organisms (Figures 6D1,D2). Overall, men significantly outnumbered women with demands for pneumonia caused by unspecified organisms (X^2^ = 13.04, *p* < 0.05). Likewise, the demands per 1,000,000 people for pneumonia caused by unspecified organisms in males were higher than that of females every year from 2015 to 2020, but no significant trend was detected (*p* > 0.05, Figure 5D2). The pre-hospital EMS demands per 1,000,000 people for pneumonia caused by unspecified organisms mainly focused on juvenile and older adult patients in Handan, and middle aged and young adults had the minimal demands per 1,000,000 people to pneumonia caused by unspecified organisms (Figure 5D3 and Supplementary Table 4). Among all the age groups (10-year intervals), only patients aged 51 to 60 years old displayed a significant decreasing percentage trend (*p* < 0.01, Supplementary Table 4).

## 4 Discussion

In this study, we described and quantified the trends and characteristics of the pre-hospital emergency care from 2011 to 2024 in Handan, and found that pre-hospital EMS demand was growing with continually enhancing capabilities during the past 14 years. In addition, the disease spectrum of pre-hospital emergency service changed markedly, and finally, the gender, age and region changes in diseases regarding pre-hospital EMS in Handan would offer scientific basis for making monitoring and prevention and control tactics.

Handan Emergency Rescue Command Center was initially established for commanding and dispatching pre-hospital emergency vehicles and personnel in 2007 (19). At the first stage of information construction, the coverage of pre-hospital EMS was mainly concentrated in main urban area and Fengfeng district of Handan. Due to the stable population of coverage areas, the pre-hospital EMS demand per 1,000,000 people in Handan fluctuated with the pre-hospital EMS demand from 2011 to 2016. Although the total number of pre-hospital EMS cases both in urban and rural areas of Handan has increased, notably, there was a marked decline in the pre-hospital EMS demand per 1,000,000 people from 2016 to 2019, which could be attribute to the increasing number of aid stations as well as the fact that Handan’s other counties (cities, districts) were embedded in the pre-hospital emergency medical system. These new emergency coverage areas in these counties (cities, districts) might exhibited decreased initial demand rates, and similar low demand rate was also seen in the Ecological Conservation Region of Beijing (1). However, when the new coverage areas were firstly integrated into the pre-hospital emergency medical system, their initial demand state could not reflect the realistic demands. With the spread of pre-hospital EMS network, demand for pre-hospital emergency care in the new coverage areas, especially the farther counties, rose steadily. We believed the aging population contributes to increased demand for pre-hospital care in these peripheral areas of Handan (2, 7). Urbanization and population aging of Handan mutually developing demographic transitions were of significant influences on the societies, which have resulted in a growing number of older adult individuals living alone, intensifying the imbalance of between the supply and demand of pre-hospital EMS in Handan (2, 16, 17, 22). Promoting early detection and screening for disease, improved social medical funding, and other unforeseen factors, may also lead to the increase in demands for pre-hospital EMS, which conferred sustained pressure on the EMS system (1, 22).

Monitoring the age trends of pre-hospital EMS patients could help the government and medical institutions to optimize EMS. Although the alarming health status of young people might be related to increased social competition and great pressure on young people to work and live (23), we still found that the proportion of people aged 21–30 and 31–40 years old in Handan was gradually decreasing from 2018 to 2024. The aging of the population has become a significant trend in China. We observed that the proportion of people aged 61–70 and >70 years old have followed increased trends in Handan. This might be explained by the effect that the older adult patients commonly experienced deterioration in physical condition, an increased prevalence of injury-related diseases, comorbidity of chronic diseases (CCD), limitations in activities of daily living (ADLs), and a reduction in daily activities (24), has posed a significant public health challenge, and should be the key objects of emergency medical services, and it was the basic guarantee for emergency medical services to develop effective pre-hospital EMS systems in retirement communities.

Here, we found the characteristics of disease spectrum changed markedly over the study period, and injury-related diseases accounted for the largest proportion of pre-hospital emergencies, which was consistent with that trauma was the most frequent diagnosis of patients transported by Helicopter Emergency Medical Services in East Azerbaijan Province, Iran (25). The demand rate for injury-related diseases of peripheral counties was relatively lower but increased rapidly and surpassed the rate in part of Handan’s urban areas, which was similar to the urban–rural disparities at the rapid development stage of urbanization in Beijing (1). To reduce the burden of injury-related diseases, besides traffic safety regulations, other preventive measures, such as public safety regulations, occupational safety standards, should be strengthened, implemented, and enforced (26). Injury-related diseases pose major public health challenges, accounting for 10% of the global burden of disease worldwide (27). In China, with the development of social economy, injury-related diseases has factored increasingly in overall mortality and disability rates, becoming the leading cause of death for persons under 45 years of age before 2015 (1, 28). However, we observed that the demand rates for injury-related diseases have followed declining trends among youth aged 21–30 years and 31–40 years in Handan, which we believed are the benefits of reducing the rate of potential years of life lost (PYLL), as younger individuals were given greater weight than older people during the calculation of the PYLL metric. Considering the traffic was the leading cause of traumatic events in China from 2012 to 2018, accounting for 52.1% of the total events (29), the merit of declining trends in injury-related diseases among younger individuals might be attributed to the conscientious implementation of the new road safety rule “one helmet, one belt” in Handan, aiming to protect people on the roads by mandating scooter riders to wear helmets and car drivers to fasten their seatbelts. Our evidence shows that the practice and effect of implementing the “one helmet, one belt” policy are of much referential value to other cities at home and abroad. Compared with adults, unintentional injury is one of the leading causes of death or disability for the children and adolescents, which accounted for 15.4% of 2.6 million deaths among children aged 1 to 14 years in the world (30). Preschool children under 6 years old in Shanghai accounted for about two-thirds of unintentional injuries in the emergency department (31). Here, we observed that the elasticity of EMS demand for injury-related diseases was kept at an extremely low level among the children aged 0–10 years from 2018 to 2024 in Handan, which meant the improvement of the living conditions, the development of healthcare, and parental supervision have provided important protections for child development in Handan. Additionally, an upward trend in injury-related diseases among older adult individuals occurred in Handan, which should be attributed to their gradual decline in physiological functions, including weakened of muscle strength, decreased balance ability, and reduced sensory organ function, diminished ability to control their surroundings and particular vulnerability to injuries (32, 33). The scientific and effective planning and allocation of medical resources and maintenance of well-established transfer guidelines for prehospital providers were crucial to reduce triage errors (34, 35). In addition, first-aid training, psychological counseling and health education directed toward residents through relevant medical institution or community committees, should focus on raising awareness of injury risk factors, conducting psychological interventions and promoting healthy lifestyles in high-risk groups if necessary (36).

In the past two decades, chronic non-communicable diseases (CNCDs) have become the leading disease burden, with cardio-cerebrovascular diseases being the main causes of death in chronic diseases. In the study, we unsurprisingly found pre-hospital EMS demand for cerebrovascular diseases increased significantly. However, it should be noted that the demand because of heart diseases remained fluctuating in Handan, which was inconsistent with the upward trend observed in some China’s megacities or super large cities, like Beijing (1) and Hangzhou (11). This might be explained by the combined effects of social and economic development (i.e., better education, higher income and better health care coverage). Given that living in more urbanized areas increases the risk of acquiring chronic diseases (37), the aforementioned situation might result from the disparities in dietary habit, stressful life events, physical activity, anxiety, living environment, and so on between Handan and these megacities/super large cities. Regional variation of the pre-hospital EMS demands for cerebrovascular diseases and heart diseases demonstrated similar spatial patterns in Handan, that is, the overall demand was higher in the urban areas than that in peripheral counties, but the demand rose at a quicker pace in peripheral counties. The urban areas were the most advanced and urbanized, rich in pre-hospital emergency resources (38), with the gradual increase of citizens’ income and the continuous improvement of health consciousness and health service system, people’s pre-hospital EMS demands for CNCDs have been released, and constantly maintained the high levels. In the past, the peripheral counties were deemed to be less economically developed and typically played a subordinate role in Handan’s economy. However, this situation has been continuously improved in recent years, with the completion of “Village to Village Roads” in Handan, the level of rural road traffic network has been comprehensively upgraded, which provided a prerequisite for the development of enclave economy, rural revitalization and urban–rural integration (39). Along with these economic strategies, “one county, one specialty” has supported the rapid and high-quality development of the peripheral counties’ characteristic economies (40), which were reflected by the fast-growing pre-hospital EMS demands for cerebrovascular diseases and heart diseases. It was noticed that suburb areas had lower density of ambulances and physicians than urban areas in Beijing, which could resulting in prolonged EMS response time and reduced survival rate in patients with cardiovascular disease (41). To meet the newly released pre-hospital EMS demands for CNCDs in peripheral counties, emergency medical resource should be rationally allocated in the future. Cerebrovascular diseases and heart disease in males were significantly higher than that of females in Handan. The gender disparity might be ascribed to both sociocultural and biological factors. Inappropriate lifestyles in particular have been implicated as significant contributors to disease severity, and gender-specific patterns are quite apparent. Both cigarette smoking and alcohol consumption rates in China are so much higher in men than in women, and the chronic problems related to these lifestyles are particularly acute in China (42). Cigarette smoking is a major risk factor for cardiovascular and respiratory diseases, over 20 different types or subtypes of cancer, and many other debilitating health conditions. The World Health Organization has stated that no level of alcohol consumption is safe for our health (43). Biologically, women possess a more robust immune response against various adverse factors than men, which may be attributed to the greater number of immune-mediated genes on the X chromosome (44). Additionally, sex hormones play a fundamental role, because the estrogens have protective roles, modulate infammatory responses and metabolism in women, which may contribute to a lower risk in infectious diseases and some chronic non-communicable diseases (45). Thus, it is important to strengthen health education and promote cardiovascular disease prevention and lifestyle modifications, including smoking cessation, alcohol reduction, and healthy dietary habits for the male population, especially for those engaged in high-risk industries and high-pressure occupations. The demand rates for cerebrovascular diseases increased significantly with increasing age, people with advance aged were those at high risk of developing cerebrovascular disease. Cardiovascular disease can serve as a prototype of chronic diseases in adulthood which showed the same pertinent basis-ageing (46). A complex health intervention (health coaching, home blood pressure monitoring, and blood pressure audit and feedback) was demonstrated to achieve a significant 33% (95% CI 27–39) reduction in the hard composite primary outcome of cardiovascular disease, heart failure, myocardial infarction and stroke death (47, 48). As for heart disease, we observed that the proportion trend of the pre-hospital EMS has followed a declining trend for the near-retirement population (aged 51–60 years old) but an expansion trend for the younger older adult population (aged 61–70 years old) in Handan. This might be explained by the effect that the combined effects of social and economic development (i.e., better education, higher income and better health care coverage) offset the effects of age on pre-hospital EMS demand among the near-retirement population, as for younger older adult population, the age effect overwhelmed these factors and ultimately increased the pre-hospital EMS demand proportion on the whole (49). Given the demands for cerebrovascular diseases and heart disease, involving multiple factors, comprehensive measures should be taken to prevent and control the occurrence of cardiovascular disease.

Although the pre-hospital EMS demands for pneumonia caused by unspecified organisms in Handan continued to rise from 2018 to 2024, it remained at a relatively low level in both the urban areas and peripheral counties. The phenomenon could be attributed to the overall improvement of health contributed by the urbanization in Handan, which drove a major shift in disease patterns towards a rise in chronic diseases (50). Men significantly outnumbered women with demands for pneumonia caused by unspecified organisms. The disparity might be ascribed to complex interactions between biological sex-related factors (e.g., hormonal and immune response variations as previously detailed) and gender-related social factors (e.g., health-related behaviors and occupational roles) (51). Men have a higher smoking rate than women. Long term smoking can lead to lung inflammation and airway damage, increasing the risk of pneumonia infection. Compared to women, men were more likely to engage in high-risk occupations such as miners, construction workers, police officers, etc., which may increase their exposure to bacteria and viruses, thereby increasing the risk of infectious diseases. The pre-hospital EMS demands for pneumonia caused by unspecified organisms mainly concentrated in juvenile and older adult patients in Handan. Children were prone to pneumonia due to their underdeveloped body and weak immune system. The older adult obtained a higher chance of developing pneumonia due to various chronic diseases, smoking, weakened immune system, and decreased mucosal barrier function in the respiratory tract. To prevent these burdens, it is crucial for the vulnerable groups to receive pneumococcal and influenza vaccinations, because the vaccinations are protective against infection for the entire population (52). Due to the world is facing two analogous megatrends that will fundamentally change the long-term emergency medical care services of its cities: urbanization and aging populations, the insights gained from this research will serve as valuable references for other Type-II large cities at home and abroad, benefiting a diverse populace and potentially contributing to long-term improvements in public health.

Here, the pre-hospital EMS demand in both the urban areas and the peripheral counties increased rapidly, emergency services could not currently meet all the patients’ needs (1, 53). In our previous study, we have showed that accessibility to pre-hospital EMS resources varied significantly across Handan and there was a notable pattern of higher accessibility in urban areas and lower accessibility in the peripheral counties, which meant that the gradient of service provision decreases with the distance from the location of central EMS resources (38). Therefore, it was urgent to establish a comprehensive and highly efficient framework of pre-hospital emergency stations based on the characteristics and trends of EMS demands including age distribution, disease spectrum, and demographic shifts. The epidemiological results revealed in this study was expected to uncover the possible weak links where pre-hospital EMS still need to be improved and enhanced and assisted in decision- makings in future EMS planning.

In the study, we have provided an integrated view of age distribution, disease spectrum, demographic shifts, and regional disparities of pre-hospital EMS demands in Handan. Base on this study, the further research could use the Dynamic Bayesian Networks (DBN) model to forecast the number of pre-hospital EMS patients in dynamic (54), facilitating the development of tailored prevention and control strategies. Additionally, by integrating multi-source big data, including traffic data, existing emergency facility data, road network data, etc., the site selection model could be used to reasonably configure the emergency medical resource in Handan (55), which could solve the problem of low efficiency in the allocation of emergency services and improve the efficiency and quality of pre-hospital emergency services.

There are some limitations for this study. First, the pre-hospital first aid data originated from Handan Emergency Rescue Command Center, there might be some potential biases in data collection, such as regional disparities in pre-hospital EMS reporting. Not all emergency patients access formal EMS services (e.g., calling 120), we could not exclude the possibility that a few patients might go directly to emergency department of hospital by private means, the unrecorded cases can lead to underestimation of EMS demand and introduce bias into the analysis of disease distribution and EMS utilization. Second, the mobility of Handan’s population compared to other cities was not discussed owing to that the detailed information of mobile population in Handan was not obtained. Population mobility and healthcare access changes could be important potential confounding factors that misinterpreted the characteristics and trends of pre-hospital emergency care in Handan, migrants may undergo immunization inequalities after migration and/or arrival (56), which underscored migration history as an independent confounding factor for the measurement of pre-hospital EMS demand. The healthcare access changes might result in an overestimation or underestimation of Handan’s pre-hospital EMS demand, due to the fact that not all people could fully access and utilize the public healthcare service. In the future, it is needed to determine these potential confounding factors. Additionally, the missing data cases are present due to the information system construction, which may cause slight inaccuracy. Data from these 7 years (2018–2024) were used to analyzing the distribution of disease characteristics.

## 5 Conclusion

Taken together, pre-hospital EMS demand in Handan has increased substantially during the past 14 years. Injury-related diseases accounted for the largest proportion of pre-hospital emergencies, and the disease spectrum of pre-hospital emergency service changed markedly. These characteristics and tendencies of different diseases revealed in this study would offer the critical steps for management and prevention of pre-hospital EMS related issues in clinical practice and public health.

## Data Availability

The raw data supporting the conclusions of this article will be made available by the authors, without undue reservation.
