# Supplementary material for: Epidemiological characteristics and trends of pre-hospital emergency care in Handan, China from 2011 to 2024
Source: Front Public Health. 2025 Sep 8;13:1651467. doi: 10.3389/fpubh.2025.1651467 (PMC12450886; doi:10.3389/fpubh.2025.1651467)
Supplement: Supplementary file 1 [file Table_1.docx]

**Supplementary Table 1. The age percentage trends of injury-related disease from 2018 to 2024.**

| **Age groups** | **2018** | **2019** | **2020** | **2021** | **2022** | **2023** | **2024** | ***p-*Value** | **Trend** |
| --- | --- | --- | --- | --- | --- | --- | --- | --- | --- |
| 0~10 | 0.02 | 0.03 | 0.03 | 0.03 | 0.03 | 0.03 | 0.03 | 0.07 | - |
| 11~20 | 0.12 | 0.11 | 0.10 | 0.11 | 0.11 | 0.10 | 0.11 | 0.76 | - |
| 21~30 | 0.12 | 0.12 | 0.11 | 0.11 | 0.10 | 0.10 | 0.09 | <0.01 | ↓ |
| 31~40 | 0.24 | 0.24 | 0.23 | 0.23 | 0.22 | 0.20 | 0.18 | <0.01 | ↓ |
| 41~50 | 0.14 | 0.14 | 0.14 | 0.14 | 0.13 | 0.14 | 0.14 | 0.55 | - |
| 51~60 | 0.18 | 0.18 | 0.20 | 0.19 | 0.20 | 0.19 | 0.18 | 0.37 | - |
| 61~70 | 0.09 | 0.11 | 0.11 | 0.11 | 0.11 | 0.13 | 0.14 | <0.05 | ↑ |
| >70 | 0.08 | 0.09 | 0.09 | 0.08 | 0.10 | 0.11 | 0.12 | <0.05 | ↑ |

**Supplementary Table 2. The age percentage trends of cerebrovascular diseases from 2018 to 2024.**

| **Age groups** | **2018** | **2019** | **2020** | **2021** | **2022** | **2023** | **2024** | ***p-*Value** | **Trend** |
| --- | --- | --- | --- | --- | --- | --- | --- | --- | --- |
| 0~10 | 0.00 | 0.00 | 0.00 | 0.00 | 0.00 | 0.00 | 0.00 | 0.55 | - |
| 11~20 | 0.01 | 0.01 | 0.01 | 0.00 | 0.00 | 0.01 | 0.00 | 0.13 | - |
| 21~30 | 0.01 | 0.01 | 0.01 | 0.01 | 0.01 | 0.01 | 0.01 | 0.37 | - |
| 31~40 | 0.06 | 0.06 | 0.07 | 0.04 | 0.04 | 0.05 | 0.03 | 0.23 | - |
| 41~50 | 0.11 | 0.07 | 0.08 | 0.07 | 0.07 | 0.08 | 0.07 | 0.55 | - |
| 51~60 | 0.27 | 0.28 | 0.29 | 0.28 | 0.25 | 0.22 | 0.18 | 0.13 | - |
| 61~70 | 0.22 | 0.23 | 0.23 | 0.23 | 0.24 | 0.24 | 0.26 | <0.05 | ↑ |
| >70 | 0.33 | 0.34 | 0.32 | 0.36 | 0.38 | 0.40 | 0.44 | <0.05 | ↑ |

**Supplementary Table 3. The age percentage trends of heart diseases from 2018 to 2024.**

| **Age groups** | **2018** | **2019** | **2020** | **2021** | **2022** | **2023** | **2024** | ***p-*Value** | **Trend** |
| --- | --- | --- | --- | --- | --- | --- | --- | --- | --- |
| 0~10 | 0.00 | 0.00 | 0.00 | 0.00 | 0.00 | 0.00 | 0.00 | 0.23 | - |
| 11~20 | 0.01 | 0.02 | 0.01 | 0.01 | 0.01 | 0.01 | 0.01 | 0.13 | - |
| 21~30 | 0.01 | 0.01 | 0.01 | 0.01 | 0.01 | 0.01 | 0.01 | 0.76 | - |
| 31~40 | 0.06 | 0.06 | 0.06 | 0.06 | 0.05 | 0.04 | 0.04 | 0.23 | - |
| 41~50 | 0.08 | 0.08 | 0.08 | 0.08 | 0.07 | 0.06 | 0.07 | 0.07 | - |
| 51~60 | 0.25 | 0.23 | 0.23 | 0.23 | 0.22 | 0.17 | 0.17 | <0.01 | ↓ |
| 61~70 | 0.17 | 0.19 | 0.19 | 0.20 | 0.21 | 0.19 | 0.20 | <0.05 | ↑ |
| >70 | 0.42 | 0.41 | 0.42 | 0.39 | 0.42 | 0.52 | 0.49 | 0.23 | - |

**Supplementary Table 4. The age percentage trends of pneumonia caused by unspecified organisms from 2018 to 2024.**

| **Age groups** | **2018** | **2019** | **2020** | **2021** | **2022** | **2023** | **2024** | ***p-*Value** | **Trend** |
| --- | --- | --- | --- | --- | --- | --- | --- | --- | --- |
| 0~10 | 0.12 | 0.09 | 0.07 | 0.14 | 0.06 | 0.03 | 0.03 | 0.07 | - |
| 11~20 | 0.01 | 0.01 | 0.01 | 0.03 | 0.01 | 0.01 | 0.01 | 1 | - |
| 21~30 | 0.00 | 0.01 | 0.03 | 0.03 | 0.01 | 0.01 | 0.01 | 1 | - |
| 31~40 | 0.01 | 0.02 | 0.04 | 0.01 | 0.02 | 0.02 | 0.01 | 0.76 | - |
| 41~50 | 0.03 | 0.02 | 0.04 | 0.02 | 0.01 | 0.01 | 0.02 | 0.13 | - |
| 51~60 | 0.15 | 0.14 | 0.15 | 0.14 | 0.12 | 0.09 | 0.07 | <0.01 | ↓ |
| 61~70 | 0.15 | 0.12 | 0.15 | 0.15 | 0.16 | 0.15 | 0.17 | 0.07 | - |
| >70 | 0.52 | 0.60 | 0.51 | 0.49 | 0.60 | 0.68 | 0.68 | 0.13 | - |
